# New Insights Into Functions and Possible Applications of *Clostridium difficile* CRISPR-Cas System

**DOI:** 10.3389/fmicb.2018.01740

**Published:** 2018-07-31

**Authors:** Anna Maikova, Konstantin Severinov, Olga Soutourina

**Affiliations:** ^1^Center for Life Sciences, Skolkovo Institute of Science and Technology, Moscow, Russia; ^2^Université Paris Diderot, Sorbonne Paris Cité, Paris, France; ^3^Microbiology, Institute for Integrative Biology of the Cell, Commissariat à l’Energie Atomique et aux Energies Alternatives, Centre National de la Recherche Scientifique, Université Paris-Sud, Université Paris-Saclay, Gif-sur-Yvette, France; ^4^Peter the Great St. Petersburg Polytechnic University, Saint Petersburg, Russia; ^5^Waksman Institute for Microbiology, Rutgers, The State University of New Jersey, Piscataway, NJ, United States; ^6^Institut Pasteur, Paris, France

**Keywords:** CRISPR, *C. difficile*, I-B subtype CRISPR-Cas system, prophage, CRISPR regulation, stress, antimicrobials, genome editing

## Abstract

Over the last decades the enteric bacterium *Clostridium difficile* (novel name *Clostridioides difficile*) – has emerged as an important human nosocomial pathogen. It is a leading cause of hospital-acquired diarrhea and represents a major challenge for healthcare providers. Many aspects of *C. difficile* pathogenesis and its evolution remain poorly understood. Efficient defense systems against phages and other genetic elements could have contributed to the success of this enteropathogen in the phage-rich gut communities. Recent studies demonstrated the presence of an active CRISPR (clustered regularly interspaced short palindromic repeats)-Cas (CRISPR-associated) subtype I-B system in *C. difficile*. In this mini-review, we will discuss the recent advances in characterization of original features of the *C. difficile* CRISPR-Cas system in laboratory and clinical strains, as well as interesting perspectives for our understanding of this defense system function and regulation in this important enteropathogen. This knowledge will pave the way for the development of promising biotechnological and therapeutic tools in the future. Possible applications for the *C. difficile* strain monitoring and genotyping, as well as for CRISPR-based genome editing and antimicrobials are also discussed.

## Crispr-Cas Systems: General Functional Aspects and Classification

The CRISPR (clustered regularly interspaced short palindromic repeats)-Cas (CRISPR-associated) systems are adaptive immune systems protecting prokaryotes against phages and other mobile genetic elements (Sorek et al., 2013). These defensive systems are found in almost all sequenced archaeal and in about half of bacterial genomes (Grissa et al., 2007). CRISPR-Cas systems are composed of CRISPR arrays and *cas* operons. CRISPR arrays in turn consist of short direct repeats (20–40 bp) separated by variable spacers. Some spacers are complementary to mobile genetic elements sequences (Shmakov et al., 2017). CRISPR arrays also contain leader regions carrying promoters from which their transcription initiates.

CRISPR-based defensive functions include two major processes: immunity (interference) and immunization (adaptation) (for general review, see Marraffini, 2015). CRISPR interference itself can be divided into two phases: the biogenesis of CRISPR RNAs and the targeting phase. At the first phase a CRISPR array is transcribed into a long RNA transcript (pre-crRNA), which is processed into small CRISPR RNAs (crRNAs), each consisting of one spacer and flanking repeat sequences. Individual crRNAs bind to Cas proteins forming a nucleoprotein effector complex, which is necessary for the second, targeting phase. The crRNAs serve as guides for recognizing nucleic acids by complementary base pairing. In this way, crRNAs direct recognition and, ultimately, cleavage of genetic elements by the Cas nucleases (Garneau et al., 2010). Spacers are incorporated into CRISPR arrays in the process of adaptation (Jackson et al., 2017). Cas1 and Cas2 proteins, found in almost all investigated CRISPR-Cas systems, are essential for this process (Koonin et al., 2017). A very important aspect of CRISPR-based immunity is the ability to distinguish host DNA from the foreign one. Protospacer-adjacent motifs (PAMs) are short sequences situated on the 3′ or 5′ end of the protospacer (foreign DNA region corresponding to a CRISPR spacer) and required for protospacer recognition. PAMs are absent from CRISPR arrays, which prevents autoimmunity (Sorek et al., 2013). PAMs are essential during spacer selection at the adaptation stage, which ensures that acquired spacers are functional in interference. Previous studies in type I CRISPR-Cas systems identified the sequence requirements for the CRISPR targeting that includes a perfect match between the 5′ end of the spacer and the protospacer within up to a 10-nt “seed” sequence (Semenova et al., 2011; Wiedenheft et al., 2011; Maier et al., 2013a).

CRISPR-Cas systems are highly diverse. This is reflected in both CRISPR array architectures and *cas* genes composition (Takeuchi et al., 2012). The variability of *cas* gene sets has formed the basis of CRISPR-Cas systems classification (Makarova et al., 2011). All investigated CRISPR-Cas systems are divided into two classes, characterized by the composition of *cas* genes involved in interference module (Koonin et al., 2017). These classes in turn are divided into six types and 33 subtypes (see **Table 1** for examples). The Class 1 comprises the most abundant and diverse type I and type III CRISPR-Cas systems as well as rare type IV. These types of CRISPR-Cas systems are found in both archaeal and bacterial genomes. Effector complexes of the type I and type III include Cas5, Cas7, Cas8 (in type I), and Cas10 (in type III) proteins (Koonin et al., 2017). For crRNA processing Cas6 family proteins are necessary in these CRISPR-Cas systems (Charpentier et al., 2015). Type I systems are also characterized by the presence of Cas3 proteins responsible for degradation of DNA recognized by effector complexes (Brouns et al., 2008). The Class 2 includes type II, type V and type VI CRISPR-Cas systems. These systems possess effector modules consisting of only one multi-domain protein. The most characterized is the type II Cas9 protein widely used in genome editing (Wang et al., 2016).

**Table 1:** Main CRISPR-Cas systems subtypes and examples of system-harboring microorganisms and clostridial species.

Class	Subtype	*cas* operon composition	Example	Examples of clostridial species and strains
Class 1	I-A	*cas6, cas11(csa5), cas7, cas5, cas8a1, cas3’, cas3”, cas2, cas4, cas1, cas4*	*Listeria monocytogenes* L99 (Sesto et al., 2014)	*C. stercorarium* subsp. *stercorarium* DSM 8532 (Poehlein et al., 2013); *C. tetani* ATCC 9441 (Cohen et al., 2017)
	I-B	*cas6, cas8b1, cas7, cas5, cas3, cas4, cas1, cas2*	*Haloferax volcanii* H119 (Maier et al., 2013b)	*C. difficile* 630, *C. difficile* R20291 (Boudry et al., 2015); *C. pasteurianum* BC1 (Pyne et al., 2016); *C. acetobutylicum* GXAS18-1 (Peng et al., 2014); *C. tetani* ATCC 9441 (Cohen et al., 2017)
	I-C	*cas3, cas5, cas8c, cas7, cas4, cas1, cas2*	*Desulfovibrio vulgaris* str. *Hildenborough* (Hochstrasser et al., 2016)	*C. cellulolyticum* H10 (Brown et al., 2014)
	I-U	*cas3, cas8u2, cas7, cas5-cas6, cas4-cas1, cas2*	*Geobacter sulfurreducens* (Koonin et al., 2017)	–
	I-D	*cas3’, cas3”, cas10d, cas7(csc2), cas5(csc1), cas6, cas4, cas1, cas2*	*Cyanothece* sp. 8802 (Koonin et al., 2017)	–
	I-E	*cas3, cas8e(cse1), cas11(cse2), cas7, cas5, cas6, cas1, cas2*	*Escherichia coli* K12 (Koonin et al., 2017)	–
	I-F	*cas1, cas2-cas3, cas8f(csy1), cas5(csy2), cas7(csy3), cas6f*	*Pseudomonas aeruginosa* PA14 (Wiedenheft et al., 2011)	–
	III-A	*cas6, cas10, cas11(csm2), cas7(csm3), cas5(csm4), cas7(csm5), csm6, cas1, cas2*	*Staphylococcus epidermidis* (Koonin et al., 2017)	*C. tetani* ATCC 453 (Cohen et al., 2017)
	III-B	*cas7(cmr1), cas10, cas5(cmr3), cas7(cmr4), cas11(cmr5), cas6, cas7(cmr6*)	*Pyrococcus furiosus* (Koonin et al., 2017)	*C. botulinum* ATCC 3502 (Negahdaripour et al., 2017)
	III-C	*cas7(cmr1), cas7(cmr6), cas10, cas7(cmr4), cas11(cmr5), cas5(cmr3)*	*Methanothermobacter thermautotrophicus* (Koonin et al., 2017)	–
	III-D	*cas10, cas7(csm3), cas5(csx10), cas11(csm2), cas7(csm7), cas7(csm5), all1473, cas7(csm5)*	*Synechocystis sp.* 6803 (Makarova et al., 2015)	–
Class 2	II-A	*cas9, cas1, cas2, csn2*	*Enterococcus faecalis* OG1RF (Bourgogne et al., 2008)	–
	II-B	*cas9, cas1, cas2, cas4*	*Legionella pneumophila* str. *Paris* (Koonin et al., 2017)	–
	II-C	*cas9, cas1, cas2*	*Neisseria lactamica* 020-06 (Koonin et al., 2017)	*C. perfringens* JGS1495 (Pearson et al., 2015)
	V-A	*cas12a(cpf1), cas4, cas1, cas2*	*Francisella cf. novicida* Fx1 (Koonin et al., 2017)	–
	V-B	*cas12b(c2c1), cas4, cas1, cas2*	*Alicyclobacillus acidoterrestris* (Koonin et al., 2017)	–
	V-C	*cas1, cas12c(c2c3)*	*Oleiphilus* sp. (Koonin et al., 2017)	–
	V-D	*cas1, cas12d(casY)*	*Bacterium* CG09_39_24 (Koonin et al., 2017)	–
	V-E	*cas12e(casX), cas4, cas1, cas2*	*Deltaproteobacteria bacterium* (Koonin et al., 2017)	–


*CRISPR-Cas systems subtypes and the composition of *cas* operons are shown according to classification of Koonin et al. (2017). Fused *cas* genes in operons are marked with a dash.*

The type I CRISPR-Cas systems are highly diverse and subdivided into seven subtypes (I-A, I-B, I-C, I-U, I-D, I-E, I-F) (Makarova et al., 2015). The subtypes I-C, I-D, I-E, I-F are encoded by a single operon in CRISPR loci, whereas subtype I-A and I-B are often encoded by several operons. I-C, I-E, and I-F subtypes are mostly present in bacteria, while I-A, I-B, and I-D are common in archaea (Makarova et al., 2011) (**Table 1**). The subtype I-B, characterized by a specific Cas8b protein, is present in methanogenic and halophilic archaea and in clostridia. Studies of the I-B CRISPR-Cas systems in haloarchaea showed some interesting features such as multiple PAMs and 9-nucleotide non-contiguous seed region (Maier et al., 2015). Although the subtype I-B was found in clostridial species it has not been well studied there yet. It is suggested that I-B CRISPR-Cas system possibly had been acquired by clostridia from archaea via horizontal gene transfer and afterward evolved independently (Peng et al., 2014). Other CRISPR-Cas systems subtypes, including I-A, I-C, III-A, III-B, and II-C, are also present in some clostridial species (**Table 1**).

## Characterization of *Clostridium difficile* Crispr-Cas System

*Clostridium difficile* (novel name *Clostridioides difficile*) is an anaerobic spore-forming bacterium, one of the major clostridial pathogens and the major cause of nosocomial infections associated with antibiotic therapy (Abt et al., 2016). During its infection cycle, this enteropathogen must cope with the presence of foreign DNA elements, including bacteriophages, in the crowded environment of the gut, and is thus expected to rely on efficient defense systems such as CRISPR-Cas to control genetic exchanges favored in its complex niche.

The first evidence suggesting the presence of active CRISPR-Cas system in *C. difficile* was obtained during deep-sequencing of regulatory RNAs in *C. difficile* (Soutourina et al., 2013). This study revealed abundant and diverse crRNAs. Active expression and processing of CRISPR loci was detected in this and a subsequent study (Soutourina et al., 2013; Boudry et al., 2015).

**FIGURE 1:**
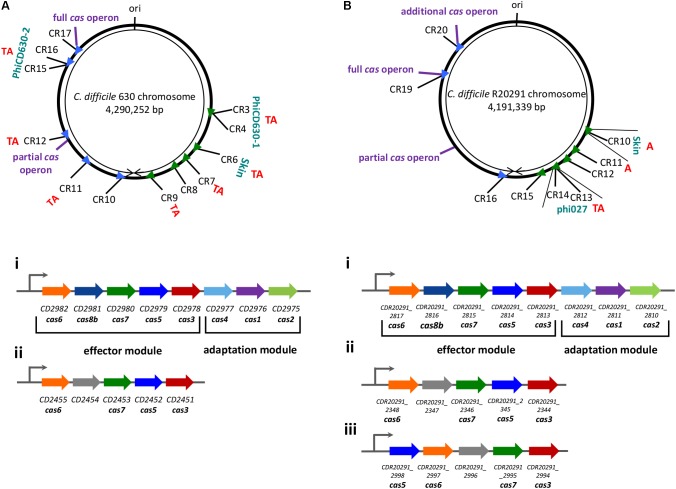
Schematic view of the chromosomal location of CRISPR arrays and the organization of the *cas* operons in *C. difficile* strains 630 **(A)** and R20291 **(B)**. CRISPR arrays (CR) are numbered according to the CRISPRdb database (Grissa et al., 2007). Arrowheads signify arrays’ position and transcriptional orientation. The locations of associated *cas* operons, prophage regions, toxin-antitoxin pairs (TA) or only antitoxins (A) and replication origin (ori) are indicated. The organization of the *cas* operons in strain 630 (left) and R20291 (right) are indicated with roman numerals, where i – full operons; ii – partial operons, iii – an additional operon. Functional modules are marked with braces. The same color was used for homologous *cas* genes (Boudry et al., 2015).

Bioinformatics analysis of more than 200 *C. difficile* genomes (Hargreaves et al., 2014; Andersen et al., 2016) demonstrated that *C. difficile* CRISPR-Cas system belongs to I-B subtype (Koonin et al., 2017). *C. difficile* CRISPR-Cas system possesses several original features (**Figure 1**). CRISPR-Cas system of this enteropathogen is characterized by an unusual large set of CRISPR arrays. For example, reference 630 and hypervirulent R20291 *C. difficile* strains contain 12 and 9 CRISPR arrays, respectively (Soutourina et al., 2013; Boudry et al., 2015). These CRISPR arrays are orientated in the direction of chromosome replication, as observed for highly expressed bacterial genes and presumably ensuring their optimal transcription (Arakawa and Tomita, 2007; Boudry et al., 2015). On average, known *C. difficile* genomes contain 8.5 arrays (Andersen et al., 2016). In most sequenced *C. difficile* strains several CRISPR arrays are located in prophages (Hargreaves et al., 2014; Boudry et al., 2015). The crRNAs originating from arrays located in prophages were found to be the most expressed in 630 and R20291 strains. Prophage localization of actively expressed CRISPR arrays may play a role in preventing infection by related competing phages by targeting their DNA (Sorek et al., 2008).

Another unusual feature of *C. difficile* CRISPR-Cas system is the presence of two or three (in 027 ribotype strains) *cas* gene sets in the majority of sequenced strains (Boudry et al., 2015) (**Figure 1**). The full *cas* operon encodes all necessary genes for CRISPR interference (*cas6, cas8b, cas7, cas5, cas3*) as well as *cas1*, *cas2*, *cas4* genes essential for spacer acquisition (Amitai and Sorek, 2016; Kieper et al., 2018; Lee et al., 2018). The additional *cas* operons lack the adaptation module. While the complete *cas* gene operons were found in ∼90% of sequenced *C. difficile* strains, the additional partial *cas* gene sets are present in almost all strains (Boudry et al., 2015). Thus, some *C. difficile* strains could have lost the ability to adapt to new genetic elements through their CRISPR-Cas systems. The *cas* operon occurrence correlates with evolutionary relationships of *C. difficile* strains reflecting their epidemiological context and, possibly, the intensity of interactions with foreign DNA elements (Boudry et al., 2015). When present, complete *cas* gene operons are usually associated with longest CRISPR arrays, which is indicative of active new spacer acquisition (or slower spacer loss) and hints to an existence of some still unknown *in cis* mechanisms responsible for different dynamics of *cas* proximal arrays. The conservation of CRISPR array structure and sequences of all CRISPR repeats in *C. difficile* genomes suggests that CRISPR arrays located far from *cas* operons use the same set of Cas proteins for their function.

An interesting evolutionary aspect of *C. difficile* CRISPR-Cas system has been recently reported (Maikova et al., 2018). Analysis of about 2,500 *C. difficile* genomes revealed co-localization of type I toxin-antitoxin (TA) modules with CRISPR arrays (**Figure 1**). TA – CRISPR array co-localization has never been reported for other bacteria and its significance remains unclear. CRISPR-arrays localized in prophage regions are in particular prone to be associated with type I TA modules, which may contribute to the stabilization of these chromosomal regions by the mechanism similar to plasmid maintenance through post-segregation killing.

The function of CRISPR-Cas system is to provide defense against viruses and other mobile genetic elements. Recent bioinformatics analysis of *C. difficile* CRISPR spacers matching known sequences showed that most of them target clostridial phages and prophage regions (Hargreaves et al., 2014; Boudry et al., 2015). This suggests that this entheropathogen actively interacts with phages, and that CRISPR-Cas actively modulates this interaction. Identification of protospacers allowed to deduce PAM sequences. While 3-nucleotide 5′-motifs CCA or CCT were most common, alternative sequences CCC, CCG, and TCA were also frequently found. Multiple PAMs were also observed in other type I-B systems (Shah et al., 2013). Conjugation efficiency experiments with plasmid vectors containing CCA and CCT PAMs and protospacers corresponding to the first spacers from actively expressed *C. difficile* 630 CRISPR arrays showed active CRISPR interference in *C. difficile* cells thus validating bioinformatically predicted PAMs and showing that *C. difficile* CRISPR-Cas system is naturally capable of defensive function (Boudry et al., 2015). Phage infection assays in 630 and R20291 strains revealed the correlation between the presence of CRISPR spacer-targeting phage sequences and phage susceptibility. Experiments using a heterologous *E. coli* host system showed that both *cas* operons of *C. difficile* 630 strain are capable of interference.

## Regulation and Potential Alternative Functions of *C. difficile* Crispr-Cas System

During its infection cycle *C. difficile* faces with different stress conditions and changing environments inside the host. To survive in phage-rich gut community while relying on the CRISPR-Cas defense, *C. difficile* needs to regulate CRISPR-Cas expression in response to different environmental signals. A study by Boudry et al. (2015) revealed that all the CRISPR arrays and *cas* genes are expressed under standard laboratory conditions. The level of CRISPR-Cas expression could be modulated by specific regulatory mechanisms.

Bacterial pathogens often form biofilms, which help them resist different threats inside the host. It was shown that *C. difficile* actively forms biofilms (Dapa et al., 2013; Nale et al., 2016; Soavelomandroso et al., 2017) during its infection cycle. Biofilm conditions are characterized by high cell densities, which increase the possibility of horizontal gene transfer (Babic et al., 2011; Abedon, 2012). Quorum sensing is one of bacterial mechanisms that regulates gene expression depending on the density of the population (Miller and Bassler, 2001). Recent studies showed that *cas* gene expression is induced by quorum sensing signals in *Serratia sp.* (I-E, I-F, and III-A subtypes) (Patterson et al., 2016) and *Pseudomonas aeruginosa* (I-F subtype) (Høyland-Kroghsbo et al., 2016). Moreover, CRISPR-Cas systems may play a role in biofilm formation and colonization of the host. For instance, CRISPR-Cas (II-A subtype) harboring *Enterococcus faecalis* strain has increased biofilm formation (Bourgogne et al., 2008). Furthermore, CRISPR-Cas-mediated gene regulation of the ability to swarm and form biofilms was revealed in *P. aeruginosa* (Zegans et al., 2009). In *C. difficile* strain 630, a recent study revealed up to 20-fold induction of *cas* genes expression in biofilms (Maikova et al., 2018), suggesting the regulation of *C. difficile* CRISPR-Cas system activity by biofilm-related factors. During infection, the complex interactions with different microbiota members within gut communities should be considered. More studies are thus needed to assess the possible link between biofilm-related signals and the regulation of CRISPR-Cas expression during the *C. difficile* infection cycle.

The obvious stress to induce CRISPR-Cas system is phage infection. At the earliest stages of attachment to the cell surface, it is often accompanied by the envelope stress (Ratner et al., 2015). The induction of the CRISPR-Cas system expression in response to this type of stress was found in different bacteria (Westra et al., 2014). Bacterial pathogens and commensals always combat with the host’s immune response, which results in a wide range of stressful effects. Several studies reported the changes of *cas* gene transcription in *Desulfovibrio vulgaris* (Mukhopadhyay et al., 2007), *Streptococcus sanguinis* (Rodriguez et al., 2011), *Pasteurella multocida* (Melnikow et al., 2008), and *Lactobacillus rhamnosus* (Koskenniemi et al., 2011) in response to different stresses such as changes in growth rate, bile, oxidative, nitrosative stresses and exposure to antibiotics. Virulence is a specific response of pathogens to different stress factors inside the host (Louwen et al., 2014). The regulation of CRISPR-Cas systems during the infection cycle may indicate an important role of these systems in pathogens. Recently, a role of an alternative SigB factor in stress response was investigated in *C. difficile* (Kint et al., 2017). Interestingly, SigB-dependent promoters were found upstream of both *cas* operons in *C. difficile* strain 630 (Maikova et al., 2018) and fivefold decrease in expression levels of both *cas* operons was observed in *sigB* mutant strain. This suggests regulation of *C. difficile* CRISPR-Cas system via stress-related signals and a potential role of this system in the survival of *C. difficile* inside the host.

Besides the adaptive immunity, multiple alternative functions of CRISPR-Cas systems have been revealed (Louwen et al., 2014; Westra et al., 2014). These functions occur through targeting bacterium’ own genes by partially or fully matching crRNAs. For instance, in *Listeria monocytogenes* a specific long type I-A CRISPR array transcript *rliB* processed by polynucleotide phosphorylase (PNPase) controls the expression of the *feoAB* genes important for virulence (Mandin et al., 2007; Sesto et al., 2014). An *rliB* mutant colonizes its host more effectively than the wild type strain. Bioinformatics analysis of *C. difficile* CRISPR spacers showed that all investigated strains carry genome-targeting spacers (Boudry et al., 2015). It may thus be speculated that *C. difficile* CRISPR-Cas system might also have functions in the regulation of the endogenous gene expression. The possible role of CRISPR-Cas systems in genome evolution via self-targeting is actively discussed (Westra et al., 2014).

## Potential Applications of *C. difficile* Crispr-Cas System

During the last decade, discoveries in the CRISPR field led to rapid development of revolutionary biotechnological applications especially in genome editing by CRISPR-Cas9 technology (Hsu et al., 2014). Different CRISPR-based tools have proved to be effective both in prokaryotes and eukaryotes (Hsu et al., 2014; Barrangou and Horvath, 2017).

Since spacers are acquired in an orderly manner, with more recently acquired spacer being closer to the leader sequence (Barrangou et al., 2007; Nuñez et al., 2015) the order of spacers within an array reflects phage invasions in different populations of the same bacterial species. This feature can reveal phylogenetic relations between strains and can be used in genotyping techniques (Louwen et al., 2014; Andersen et al., 2016). Such “CRISPR-typing” has been already applied for outbreak tracking of *Yersinia pestis* (Cui et al., 2008; Barros et al., 2014) and *Salmonella enterica* (Timme et al., 2013; Pettengill et al., 2014). Moreover, CRISPR typing is capable to reveal antibiotic-resistant phenotypes (Palmer and Gilmore, 2010) or prophages (Nozawa et al., 2011). These correlations can be explained by the influence of active CRISPR-Cas systems on the horizontal gene transfer, which plays important role in the acquisition of new genes and operons, essential for bacterial pathogenesis and adaptation (Louwen et al., 2014). CRISPR-typing approach based on spacer content and polymorphism can be successfully applied to *C. difficile* with correlation between CRISPR-groups and toxin groups (Andersen et al., 2016).

CRISPR-Cas systems can be applied for development of new antimicrobials based on the self-targeting (Bikard et al., 2012). The general strategy is the use of phage particles and phagemids as vectors to deliver auto-targeting CRISPR-Cas components inside a pathogenic cell (Bikard and Barrangou, 2017). Many pathogens possess endogenous active CRISPR-Cas systems, which can be repurposed for self-targeting. Since *C. difficile* contains a naturally active CRISPR-Cas system, such a strategy could be promising for control and even treatment of *C. difficile* infection (CDI), in the context of recent worldwide emergence of antibiotic-resistant *C. difficile* strains (Banawas, 2018). Phage therapy of CDI has proved to be another promising alternative, but it faces some difficulties including lack of appropriate phages (Hargreaves and Clokie, 2014; Sekulovic et al., 2014). The presence of active CRISPR-Cas system should effectively prevent infection by at least some phages complicating matters further.

The most popular biotechnological application of CRISPR-Cas systems is genome editing (Barrangou and Horvath, 2017). In prokaryotes, the most interesting is the application of endogenous CRISPR-Cas systems since it requires the introduction of less additional components for the editing process. Several works showing the applications of endogenous I-B subtype systems for genome editing were recently published. The first one, by Pyne et al. (2016) describes this approach in *Clostridium pasteurianum*. In this study, a plasmid vector containing an artificial CRISPR array with a protospacer targeting the gene of interest and arms for homologous recombination was used to delete the *cpaAIR* gene encoding a restriction enzyme (Pyne et al., 2016). This approach allows fast and markless deletion or modification of the genes of interest in bacteria. Later, other studies confirmed the efficiency of this method in other I-B subtype-carrying organisms: archaeon *Haloarcula hispanica* (Cheng et al., 2017) and butanol producing *Clostridium tyrobutyricum* (Zhang et al., 2018). Another study revealed that *Haloferax volcanii* CRISPR-Cas system with deletions of *cas3* and *cas6* genes can be used for programmable repression of genes in this archaeon (Stachler and Marchfelder, 2016). Many efficient approaches for *C. difficile* genome manipulation exist to date. ClosTron is a method based on altered type II intron, which is able to insert in almost every region of the chromosome (Kuehne et al., 2011). Another method is CodA allele exchange technique based on semi-suicidal vector carrying the *E. coli codA* gene as a counter-selectable marker (Cartman et al., 2012). Successful application of CRISPR-Cas9 (McAllister et al., 2017; Wang et al., 2018) and Cpf1 (Hong et al., 2018) systems for genome editing in *C. difficile* was recently reported and may further extend our ability to manipulate the genome of this pathogen.

Despite the recent insights, many aspects of *C. difficile* CRISPR-Cas system remain to be characterized. We hope that future studies will shed new light on the secrets of *C. difficile* success within host environments relying on effective defense systems and will lead to promising medical and biotechnological applications.

## Author Contributions

AM wrote the draft of the paper. OS and KS designed the project and performed critical revisions of the manuscript.

## Conflict of Interest Statement

The authors declare that the research was conducted in the absence of any commercial or financial relationships that could be construed as a potential conflict of interest.
